# Missouri State Dental Association

**Published:** 1890-08

**Authors:** 


### Missouri State Dental Association.

St. Louis, Mo., July 29—90.

The twenty-sixth annual meeting was held at Pertle Springs, Warrensburg, Mo., July 8, 9, 10, 11, 1890. President Dr. Henry Fisher called the meeting to order.

The committee on resolutions to the memory of Dr. A. Noland, presented the following which were adopted by the association:

TO THE MEMBERS OF THE MISSOURI STATE DENTAL ASSOCIATION.

Whereas, Death has removed from our ranks our beloved brother and co-laborer, Dr. A. Noland, of Monroe City, on the 22nd of January, 1890; and

Whereas, Dr. Noland was a faithful student, an honored laborer, and worked hard to raise the standard of our profession in this State;

*Therefore, Resolved*, That this association mourn with sorrow the loss sustained.

*Resolved*, That this association hereby tender his bereaved family its heartfelt sympathies and condolence in this their sad bereavement, and may that God, in whom he so implicitly trusted, speak peace to their sad hearts in their distress.

*Resolved*, That a copy of these resolutions be sent to the family of our deceased brother and to the dental journals for publication.

B. Q. Stevens,
G. M. Risley,
Jas. L. Leavel, Committee.

The committee to draft resolutions on the death of Dr. Judd presented the following, which were adopted:

Whereas, The recent death of Dr. Homer Judd brings to mind his activity and influence in the organization of this association, and his subsequent labor in extending its usefulness, to the great benefit of the profession in this State; and,

Whereas, His noble character, energy, and literary attainments entitle him to an exalted place on the scroll of deceased members; therefore,

*Resolved*, That in the death of Dr. Judd this association has lost an honored member, whose professional character and example we emulate, and whose memory we ever hold dear.

*Resolved*, That the heartfelt sympathy and condolence of this association is tendered the family of our departed brother in their sad bereavement.

*Resolved*, That a copy of these resolutions be sent to the family and to the dental journals for publication.

W. H. EAMES,
C. W. SPALDING,
J. C. GOODRICH, Committee.
PERTLE SPRINGS, JULY 9TH, 1890.

Drs. Price, T. W. Reed, and F. Swap were appointed a committee to draft suitable farewell resolutions to Dr. Spalding, and reported as follows:

MR. PRESIDENT AND MEMBERS OF THE MISSOURI STATE DENTAL ASSOCIATION:

*Gentlemen*: We, your committee appointed to draft resolutions expressive of the pleasure of this association, afforded by the presence of Dr. C. W. Spalding, and of regret that he will soon sever his social connections with us, most respectfully submit the following resolutions:

*Resolved*, That with a full appreciation of Dr. Spalding's virtues as a gentleman, his high moral character as a man, his eminent qualifications and invaluable counsel as a professional brother, we tender to him our most sincere thanks for his presence at this meeting (probably the last time we shall all meet him on this earth); we deeply regret that he should feel it necessary to sever his social connection with us.

*Resolved*, That we fully realize the fact that no one has done more than he to advance the standard of our profession and the best interests of this association, which will ever, by us, be remembered and cherished with greatful hearts.

*Resolved*, That in his departure he takes with him our most earnest wishes for his success, prosperity, and happiness; and may kind Providence ever watch over, guide and shield him..

*Resolved*, That these resolutions be spread upon the records of this association, a copy properly engrossed be presented to Dr.

Spaulding, and one sent to each of the dental journals for publication.

JAS. A. PRICE,
T. W. REED,
F. SWAP, Committee.

The election of officers resulted as follows: President, Dr. J. F. McWilliams, Mexico; Vice-President, Dr. George L. Shephard, Sedalia; Second Vice-President, Dr. W. H. Buckley, Liberty; Recording Secretary, Dr. John G. Harper; Corresponding Secretary, Dr. William Conrad; Treasurer, Dr. James A. Price, Weston; Board of Censors, Drs. J. G. Hollingworth, W. L. Reed, Chas. L. Hungerford; Committee on Ethics, Drs. N. H. Gaines, C. V. Huff, J. W. Aikin; Publication Committee, Drs. E. E. Shattuck, H. S. Lowry, W. E. Tucker; Law, Jas. A. Price, Weston; Committee on New Appliances, Dr. J. M. Austin, St. Joseph.

Executive Committee, Dr. William Conrad, Dr. Henry Fisher, and Dr. J. W. Whipple, St. Louis.

Supervisor of Clinics, Dr. A. J. Prosser, St. Louis.

Next place of meeting, Louisiana, Mo., first Tuesday after July 4th, 1891.

321 N. Grand Av. WM. CONRAD, Cor. Sec.

---

DOCTORS AND WINE.—Nothing indicates more plainly the healthful advances in regard to diet than the changes that have occurred in physicians themselves. Two hundred years ago, and even much later, doctors were notorious for their eating and tippling, and were generally very fat. Dr. Beddoes was so stout that the ladies called him their walking feather bed, and Dr. Fleming weighed 291 pounds until he reduced his weight by abstinence and eating a quarter of an ounce of Castile soap every night, and Dr. Cheyne weighed 384 pounds. It is said that it was during the seventeenth century when doctors drank so heavily, that it became fashionable for them to write such inelligible prescriptions, which were the result of their trembling hands. The man who remains abstemious where no liquor is to be had, does not deserve much credit, but the man who is temperate when the sparkling champagne stands beside his plate merits our approbation.

